# Membrane Technology for Valuable Resource Recovery from Palm Oil Mill Effluent (POME): A Review

**DOI:** 10.3390/membranes15050138

**Published:** 2025-05-02

**Authors:** Que Nguyen Ho, Woei Jye Lau, Juhana Jaafar, Mohd Hafiz Dzarfan Othman, Naoko Yoshida

**Affiliations:** 1 Department of Civil Engineering, Nagoya Institute of Technology (Nitech), Nagoya 466-8555, Japan; nguyen.ho.que.j6@f.mail.nagoya-u.ac.jp; 2 Advanced Membrane Technology Research Centre (AMTEC), Faculty of Chemical and Energy Engineering, Universiti Teknologi Malaysia, Skudai 81310, Johor, Malaysia; lwoeijye@utm.my (W.J.L.); juhana@petroleum.utm.my (J.J.); hafiz@petroleum.utm.my (M.H.D.O.)

**Keywords:** palm oil mill effluent, water reclamation, biogas, microbial fuel cell, membrane

## Abstract

Palm oil mill effluent (POME), a byproduct of palm oil processing, has substantial resource recovery potential. Its rich biodegradable content supports methane (CH_4_) production via anaerobic digestion, enabling renewable energy generation. Additionally, the significant water content of POME can be reclaimed for use in boiler feed, irrigation, and drinking water. However, selecting appropriate technologies to recover valuable resources from POME is challenging, particularly for the purification and upgrading of biogas. Membrane technologies offer an effective approach for transforming POME treatment from an energy-intensive process into a resource recovery system, supporting the decarbonization of palm oil production and advancing global sustainability objectives. This technique is cost-effective and ecofriendly for biogas purification and water reclamation. For biogas purification and upgrading, membrane systems offer the lowest capital and operational costs at 5.654 USD/m^3^, compared to other technologies, such as 6.249 USD/m^3^ for water scrubbers and 6.999 USD/m^3^ for chemical absorbers. This review primarily explores the potential of membranes for gas purification from POME and examines their integration with other processes to develop advanced systems, such as ultrasonicated membrane anaerobic systems and membrane anaerobic systems, to enhance biogas production. In addition, water reclamation from POME is discussed, with ultrafiltration membranes emerging as the most promising candidates. Proton exchange membranes, such as Nafion, are used extensively in microbial fuel cells to improve electricity generation, and this is also summarized. Finally, challenges and future perspectives are highlighted, emphasizing the broader potential of membrane technology in POME wastewater resource recovery.

## 1. Introduction

Palm oil mill effluent (POME) is the primary biomass generated during the sterilization, extraction, and clarification of palm oil mills [1]. Studies have shown that POME pollution is 100 times higher than that of municipal wastewater [2], posing considerable disposal challenges [3]. POME consists of various suspended materials in the form of colloidal suspensions, primarily composed of water (95–96%) and a small fraction of solid matter, including total solids (TSs) (4–5%), suspended solids (SSs) (2–4%), and trace amounts of oil (0.6–0.7%) [4]. In addition, POME contains a high concentration of organic matter (OM) [5], which causes severe pollution that harms water bodies and ecosystems [6,7]. The general physicochemical characteristics of POME in Malaysia are listed in Table 1. However, they can vary depending on the quality of the raw material, operational duration, treatment methods, and season [8]. Typically, during peak crop seasons, processing plants operate at full capacity, causing POME flow rates to double compared with that in low seasons. In addition, POME becomes denser and more concentrated at significantly higher parameter levels [9]. The treatment of POME is a major source of greenhouse gas (GHG) emissions. In Malaysia, the anaerobic digestion of POME generates approximately 796,346 t of CH_4_ annually, accounting for 6.8–7.5% of the country’s total GHG emissions [10].

Despite its environmental challenges, POME has notable potential sustainable applications [22]. Its high OM content enables the production of approximately 28 m of biogas per 1 m of POME [23]. Residual oil can be converted into biodiesel via transesterification [24,25,26], and essential nutrients such as potassium (K), phosphorus (P), and nitrogen (N) make it a viable alternative biofertilizer [27]. Additionally, POME is a cost-effective substrate for microbial cultivation [28] and a source of value-added chemicals, such as enzymes, organic acids, carotenoids, phenolics, and bioflocculants/surfactants [29]. Hence, transforming POME treatment into a resource recovery system enhances its sustainability, reduces its environmental impact, and creates economic opportunities for palm oil mills.

Biogas production through anaerobic digestion is a key use of POME, with significant environmental benefits, economic potential, and solutions for energy and waste management [30,31]. This biogas typically consists of 35–75% methane (CH_4_), 25–65% carbon dioxide (CO_2_), 1–5% hydrogen (H_2_), and small amounts of ammonia, hydrogen sulfide (H_2_S), halides, and water vapor [32]. However, biogas impurities, primarily CO_2_ and H_2_S, pose challenges. High levels of CO_2_ reduce the calorific value (CV) of CH_4_ and occupy space in storage and transportation systems without contributing to their combustion efficiency [31]. Additionally, H_2_S restricts biogas use for power generation or biomethane upgrading due to equipment corrosion [33,34]. Hence, removing or reducing CO_2_ and H_2_S concentrations during POME treatment is crucial for optimizing the biogas’s quality for these applications.

In addition to biogas, microbial resource recovery can be used for electricity generation and water reclamation. POME consists of 94–96% water [35], and the production of 1 t of crude palm oil (CPO) requires 5–7.5 t of water, with over 50% turning into POME. Adopting water reclamation techniques allows POME to serve as a valuable source of recycled freshwater, promoting resource optimization and sustainability. Its high organic content also supports electricity generation through microbial fuel cells (MFCs) that convert OM into electricity during wastewater treatment [36]. This approach mitigates the environmental impact of POME, provides sustainable energy for palm oil mills, and lowers reliance on external power grids and fossil fuels, thereby supporting sustainable practices and a circular economy.

Several technologies have been developed to purify and upgrade biogas and facilitate water reclamation from POME. These include water scrubbing, absorption methods, and membrane technologies [37], which enable biogas to be upgraded to biomethane with a CH_4_ concentration of 90% or higher, comparable to that of natural gas. The use of biomethane (>90% CH_4_) is projected to reduce GHG emissions by 60–80% compared to gasoline [38]. Membranes are crucial for water reclamation and gas separation processes, including biogas upgrading, hydrogen purification, and CO_2_ capture [39,40]. They are recognized for their efficient separation, low carbon footprint, and minimal energy use [40,41]. Advancements in membrane materials have also improved the performance of MFCs in electricity generation during POME treatment [42].

While membrane technology shows promise for biogas purification, water reclamation, and the enhancement of MFC performance in the context of electrolyte membranes, limited literature exists on its application in addressing issues related to POME. Previous studies have primarily focused on their use in recovering valuable resources from wastewater. To the best of our knowledge, this is the first review to specifically focus on purifying POME-derived biogas and utilizing MFCs to generate electricity from POME. This review consolidates the existing studies, highlights the potential of membrane technologies, and outlines their advantages, such as a high separation efficiency and scalability, for upgrading bio-CH_4_ or Bio-CNG, while improving MFCs’ power density under POME-specific conditions. By providing a comprehensive overview of this field, this review aims to advance membrane-based purification systems, promote cleaner energy production, and support the circular economy for the palm oil industry.

## 2. Purification of Biogas from POME

### 2.1. Biogas Purification Mechanism Using Membranes

**Figure 1:**
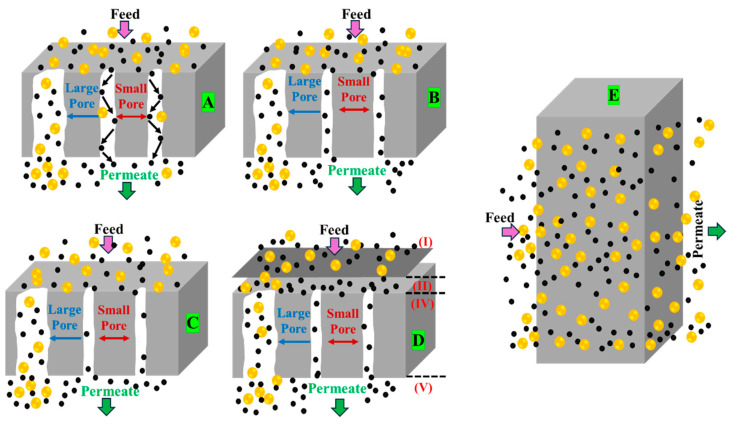
Gas separation mechanisms through porous and non-porous membranes are illustrated. Letters (**A**–**D**) represent the separation mechanisms in porous membranes, whereas (**E**) corresponds to the non-porous membrane. Each letter shows a distinct mechanism: (**A**–**E**) refer to Knudsen diffusion, molecular sieving, capillary condensation, surface diffusion, and solution–diffusion separation, respectively. I, II, III, and IV represent the stages of positioning, adsorption, diffusion, and desorption, in the surface diffusion process, respectively. Adapted with permission from [48].

Membranes for gas purification from wastewater, such as POME, are categorized as non-porous or porous [43]. Non-porous polymer-based membranes have a dense structure that enables gas permeation via pressure, concentration, or electrical potential gradients. Porous membranes, which are typically inorganic membranes, separate gases based on their molecular sizes through small pores. The key separation mechanisms include Knudsen diffusion, molecular sieving, surface diffusion, capillary condensation, and solution–diffusion [44,45,46,47], as illustrated in Figure 1.

In non-porous membranes, gas transport follows a solution–diffusion mechanism [43] as shown in Figure 1E. This process consists of three sequential steps. (1) The gas dissolves into the membrane on the upstream side, owing to a partial pressure gradient governed by the solubility of the gas in the membrane polymer [49]. (2) The dissolved gas diffuses through the polymer matrix along a concentration gradient, which is influenced by the molecular size and membrane-free volume [50]. (3) The gas desorbs downstream, thus completing this process [43]. This mechanism is essential for gas separation and carbon capture processes. Equation (1) describes the solution–diffusion model used to evaluate the flux, J (mol/m^2^·s), of gas molecules.

(1) $$J=\frac{D\times S}{l}(p_{f}-p_{p})$$

where D is the diffusion coefficient of the gas (cm^2^/s), S is the solubility coefficient of the gas, (cm^3^(STP)/cm^3^·cmHg), l is membrane thickness (cm), $p_{f}$ is the feed-side partial pressure of the gas (cmHg or atm) and $p_{p}$ is the permeate-side partial pressure of the gas (cmHg or atm).

Gas transport in porous membranes occurs as the gas moves through the pores from high to low pressure (Figure 1A–D). The separation mechanisms primarily depend on the pore size: capillary condensation (~0.1–10 μm), Knudsen diffusion (< 0.1 μm), and molecular sieving (5–10 Å) [51]. Knudsen diffusion occurs when gas molecules pass through membrane pores smaller than their mean free path, resulting in more frequent collisions with pore walls than intermolecular collisions [52]. The molar flux, J (mol/m^2^·s), due to Knudsen diffusion through a membrane can be described as Equation (2)

(2) $$J=\frac{\varepsilon }{\tau }\frac{1}{l}D_{K}\left(\frac{d_{p}}{d_{x}}\right)$$

in which ε is membrane porosity (dimensionless), τ is the tortuosity factor (dimensionless), l is membrane thickness (m), D_K_ is the Knudsen diffusion coefficient (m^2^/s), and $\frac{d_{p}}{d_{x}}$ is the pressure gradient across the membrane.

Capillary condensation occurs when gaseous compounds adsorbed in the inner pores condense on the membrane, allowing soluble gases in the condensed phase to flow from the high-pressure side to the low-pressure side [53]. The capillary entry pressure is influenced by various geometrical factors, such as membrane thickness, surface roughness, and pore shape [54]. The Kelvin equation is the main equation used to describe capillary condensation, as shown in Equation (3).

(3) $$In\left(\frac{p}{p_{0}}\right)=\frac{2\gamma V_{m}}{rRT}$$

where p is the equilibrium vapor pressure inside the pore (Pa or atm), $p_{0}$ is the saturation vapor pressure of the bulk liquid (Pa or atm), γ is the surface tension of the liquid (N/m), $V_{m}$ is the molar volume of the liquid (m^3^/mol), r is the effective pore radius (m), R is the universal gas constant (8.314 J/mol·K), and T is the absolute temperature (K). Because capillary condensation is generally governed by the Kelvin equation, incorporating its effects into broader transport models is necessary to establish its relationship with gas flux through a membrane.

Molecular sieving occurs when the pore diameters closely match the sizes of the molecules, causing molecular sieves, with pore sizes at the molecular scale, to replace Knudsen diffusion as the dominant transport mechanism. Molecular sieving selectivity is based on molecular size, with smaller molecules exhibiting higher diffusion rates [53]. In general, molecular sieving flux, J (mol/m^2^·s), can be expressed as Equation (4)

(4) $$J=\frac{D_{0}\times S}{l}(c_{f}-c_{p})$$

where $D_{0}$ is the intrinsic diffusivity of the gas in the membrane (m^2^/s); S is the steric hindrance factor or partition coefficient; l is the membrane thickness (cm); and $c_{f}$ and $c_{p}$ are the concentrations on the feed and permeate sides, respectively.

**Figure 2:**
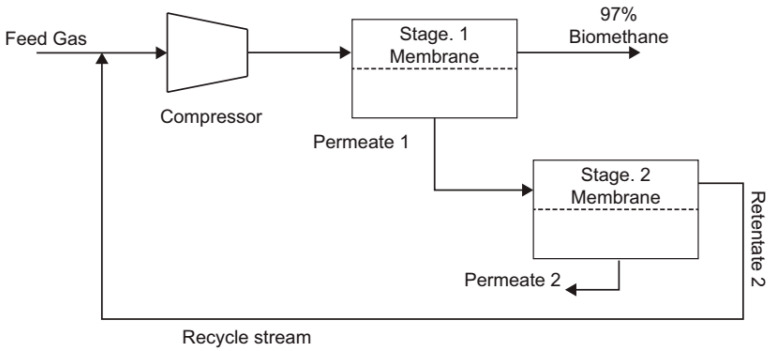
Two-stage membrane process for biogas purification. Reprinted from [55], licensed under CC BY-NC-ND 4.0.

To improve biogas upgrading via membrane separation, a two-stage system was introduced, as illustrated in Figure 2 [55]. In this system, the permeate from the first stage flows into the second-stage membrane to further recover the methane (CH_4_). The retentate from the second stage is recirculated to the first stage to ensure efficient methane recovery, while progressively eliminating CO_2_ and other impurities. This recirculation of the second-stage permeate enhances methane recovery without requiring additional compression, thus optimizing energy consumption [55,56]. There was no compression between the stages, and the pressure of permeate 1 was controlled using a flow valve at retentate 2. The pressure of permeate 1 was maintained at a constant value at a specific feed pressure [55]. To achieve the highest permeate purity, it is essential to simultaneously maximize the pressure ratio in both stages by setting the interstage retentate, as described in Equation (5).

(5) $$P_{interstage}=\sqrt{P_{feed}\cdot P_{permeate\;2}}$$

where $P_{feed}$ is the pressure of the feed gas entering the first membrane stage, $P_{permeate\;2}$ represents the permeate pressure in the second membrane stage, and $P_{interstage}$ is the intermediate pressure between the two membrane stages.

### 2.2. Study on the Use of Membranes for Biogas Purification from POME

**Figure 3:**
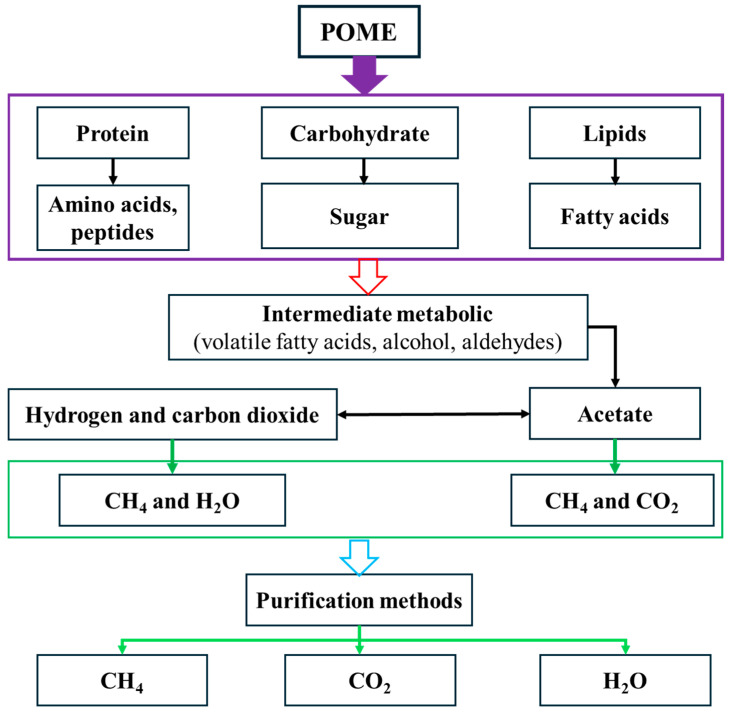
Conversion processes, from the generation of mixed biogas to its upgrading into biofuel. Adapted with permission from [57].

POME is rich in OM and is biodegradable, rendering it an ideal feedstock for anaerobic digestion to produce biogas. During anaerobic digestion, POME produces CH_4_, CO_2_, and H_2_O. The conversion process involves four stages: hydrolysis, fermentation (acidogenesis/acetogenesis), and methanogenesis (Figure 3) [57]. After the conversion of OM, the main components of the biogas are CH_4_ and CO_2_, which account for approximately 65% and 32%, respectively, with trace amounts of other gases, such as H_2_S. This gas composition is comparable to that of other feedstocks, such as municipal wastewater [58]. However, due to POME’s higher organic content and biodegradability (see Table 1), it typically generates greater gas yields, particularly CH_4_, during anaerobic digestion. Despite this potential, CH_4_ production from POME is still not fully optimized, as many mills favor open anaerobic digesters for their lower costs and simpler operation over more efficient closed systems. Therefore, capturing and purifying biogas in this sector is crucial, not only for energy recovery but also for reducing CH_4_ and CO_2_ emissions. Because these gases are potent greenhouse gases (GHGs), their release into the atmosphere contributes significantly to global warming [59]. It has been reported that the global CH_4_ potential of POME is approximately 600 million m^3^/year [59], and that CH_4_ is more effective than CO_2_ in trapping heat in the atmosphere on a per-molecule basis. Therefore, recovering CH_4_ from POME is an environmentally and economically viable solution to addressing waste management challenges while fostering renewable energy generation. This approach is consistent with global sustainability goals, reduces GHG emissions, and provides substantial cost savings for the palm oil industry [13].

**Figure 4:**
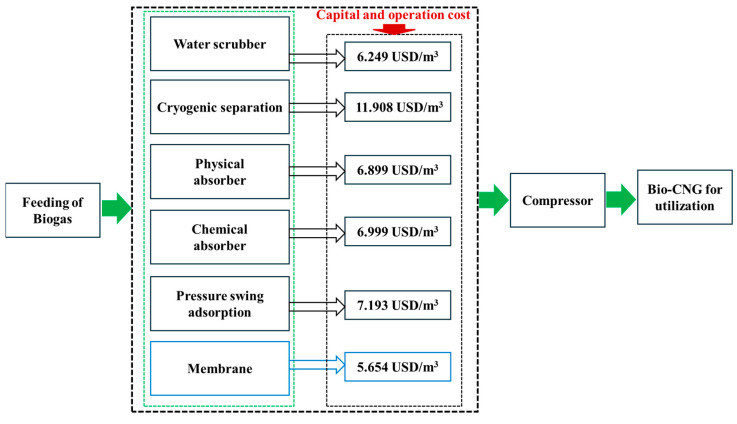
Comparison of the costs of biogas purification and upgrading technologies reveals that membrane membranes have the lowest capital and operational expenses. The figure was constructed based on data reported in [62].

**Figure 5:**
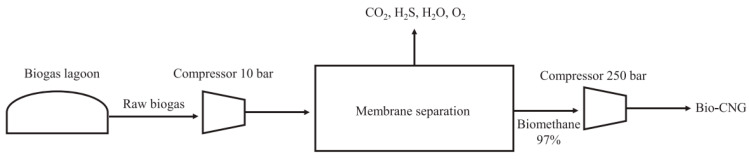
Biogas production and its purification process for bio-CNG conversion. Reprinted from [60], licensed under CC BY-NC-ND 4.0. In addition to biogas lagoons, biogas can also be produced using systems such as the continuous stirred tank reactor (CSTR), expanded granular sludge blanket (EGSSB), up-flow anaerobic sludge blanket (UASB), anaerobic filters (AF), and integrated anaerobic–aerobic bioreactor (IAAB) [61].

Membrane technology has emerged as a potential method for purifying the biogas generated from POME. This approach uses selective membranes that can effectively separate CH_4_ from impurities, such as CO_2_, H_2_S, and water vapor. Additionally, membrane systems provide advantages, such as a lower energy consumption, compact design, scalability [60], and minimal chemical usage, compared to other purification methods such as water scrubbing, cryogenic separation, and physical, chemical, and pressure swing adsorption, as shown in Figure 4. Previous studies have demonstrated that membranes can remove up to 99% of CH_4_ from biogas, thereby yielding a product with a CH_4_ purity of 96.5%, which is more efficient than the aforementioned purification methods [61]. Consequently, membrane technology, which is highly efficient and cost-effective, has emerged as a potential solution for biogas purification [62]. The process of upgrading biogas to bio-CNG using membranes is shown in Figure 5 [60], and this can be used to purify biogas derived from POME.

Although membrane technology shows potential for purifying biogas derived from POME, there is insufficient research on its application for this specific purpose. This is because POME typically contains impurities, such as CO_2_, H_2_S, and water vapor, which are challenging to separate. Additionally, because POME is primarily produced in palm oil-producing countries such as Malaysia and Indonesia, these regions may focus more on immediate solutions to reduce environmental impacts, such as biological methods for lowering chemical oxygen demand (COD) and biological oxygen demand (BOD), as well as cost-effective approaches, rather than on advanced purification technologies, which are emerging subjects. However, only a few studies have addressed these challenges. In 2020, Nasrin et al. successfully used a commercial membrane purchased from Air Products (USA) to purify biogas derived from POME, resulting in the production of bio-CNG. In their study, the team separated H_2_S from raw biogas through a combined biological and physical process using absorption in a bioscrubber, followed by activated carbon. The results show removal efficiencies of approximately 99% and 88% for H_2_S and CO_2,_ respectively. The purified bio-CNG contained 92% CH_4_, 7% CO_2_, and 0.9% O_2_, and the H_2_S concentration was reduced to 5 ppm. This system increased the CV of biogas from 20.0 MJ/m^3^ to 35.0 MJ/m^3^. The findings from this study demonstrated that after purification, the biogas derived from POME attained a quality similar to that of natural gas, considering vital components such as CH_4_, hydrocarbons, CO_2_, H_2_S, O_2_, pressure, CV, and specific gravity [63].

In another approach, a pilot-scale separation plant was constructed to upgrade biogas to bio-CNG fuel quality, with a capacity of 30 m^3^/h [64]. This system operated under two main processes: (1) a pretreatment process to remove trace components such as H_2_O and H_2_S to meet fuel standards, and (2) a membrane process using spiral-wound membranes to separate the CO_2_. In the first process, ethylenediaminetetraacetic acid (EDTA)/Fe (III) was used to remove the H_2_S-containing biogas, thereby achieving an H_2_S removal efficiency of over 99%. In the second process, single- and double-membrane modules (MTR; Membrane Technology & Research, Newark, NY, USA) were tested to compare the biogas purity. The results show that the double-stage separation configuration successfully produced biogas with over 98% CH_4_ and low CO_2_ and H_2_S concentrations (Table 2) [64]. The study demonstrated that CH_4_ recovery and separation efficiency increased when the feed temperature was reduced from 38 °C to 32 °C., consistent with previous studies [65]. For instance, with a flow rate of 7.2 m^3^/h, the CH_4_ purity at 38 °C and 32 °C was 97.8% and 99.3%, respectively. This was attributed to the higher temperatures allowing a larger volume of gas to flow through the membrane, thereby reducing the CH_4_ purity. In contrast, the CH_4_ separation efficiency increased with a higher feed pressure but decreased as the feed flow rate increased [64].

Wahyudhie et al. (2024) developed polysulfone (PSf) membranes to separate CO_2_/CH_4_ gases from POME. The results demonstrated that increasing the PSf concentration from 18% to 22% by weight enhanced the CO_2_/CH_4_ selectivity from 1.65 to 3.47. The improvement in separation performance was attributed to changes in the membrane thickness and pore size as the polymer concentration in the casting solution was adjusted. Gas permeation tests showed that the CH_4_ content in the purified biogas reached 77% by volume [66].

**Figure 6:**
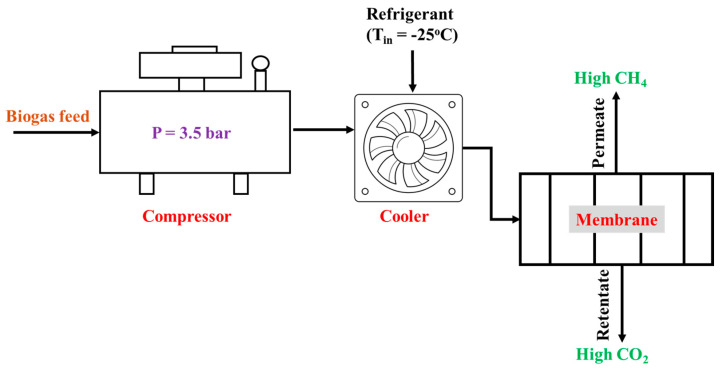
Schematic diagram of a single-stage membrane system used in the simulation process for comparing different technologies in biogas purification applications. Adapted with permission from [67].

In another approach, the use of membranes for POME purification was evaluated through simulations and compared with methyldiethanolamine (MDEA) and diethanolamine (DEA) absorption systems. Specifically, Junaidi et al. used Aspen Plus 8.0 to simulate the membrane separation process for POME under steady-state conditions [67]. For the simulation, a membrane model was developed using feed biogas containing CH_4_ and CO_2_ in the ranges of 60–70 vol.% and 30–40 vol.%, respectively, with a constant flow rate of 250 Nm^3^/h. A single-stage membrane configuration was established, as shown in Figure 6. In this study, a membrane module with a length of 15–20 m and a width of 3 m was used for the simulation [67], which corresponded to effective surface areas of 45 m^2^ and 60 m^2^, respectively. The results demonstrated that an effective membrane area of 60 m^2^ was optimal for purifying biogas from POME, as it met the sales gas standard requirement of 96% moles of CH_4_. Although the membrane process achieved a lower purity than the DEA and MDEA absorption systems, an economic analysis showed that the membrane process was more cost-effective, with an estimated payback recovery cost of approximately 0.0046 million USD/year, whereas the DEA and MDEA systems had costs of 0.153 and 0.162 million USD/year, respectively [67].

**Figure 7:**
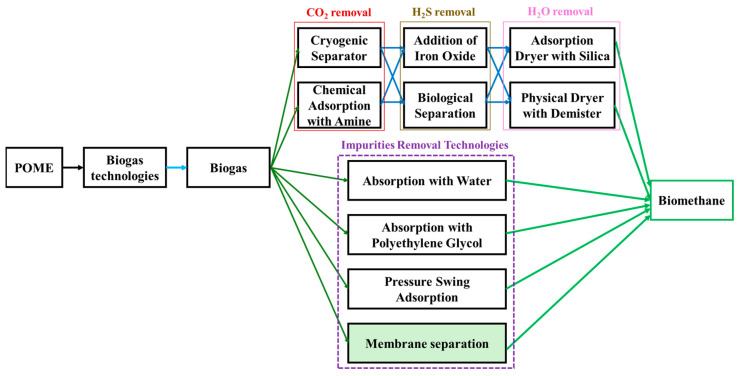
Processes for optimizing bio-CH_4_ production from POME. Adapted from [61], licensed under CC BY 4.0.

In a similar approach, a recent study used a mathematical model to evaluate the efficiency of membrane-based biogas purification from POME in comparison with other technologies, such as water, polyethylene glycol, and pressure swing adsorption. The authors proposed a process for purifying CH_4_ for bioenergy production, as shown in Figure 7 [61]. To assess the effectiveness of different technologies for sustainable bio- CH_4_ production from POME, four scenarios were simulated: (1) maximizing bio-CH_4_ production, (2) minimizing costs, (3) reducing GHG emissions during bio-CH_4_ production, and (4) integrating these scenarios [61]. The simulation results showed that the highest bio-CH_4_ production occurred after an up-flow anaerobic sludge blanket (UASB) under thermophilic conditions, with a selected temperature of 70 °C. The generated biogas was purified via membrane separation to achieve 96.5 wt% CH_4_ purity. The study revealed that the electricity consumption was 0.39 kW/kg, which is lower than that of other technologies [61]. These findings are consistent with those of a previous study that emphasized the purification of CH_4_ from biogas to upgrade it for bioenergy use [68].

## 3. Hybrid Membrane Systems Enhance POME Treatment Efficiency and Biogas Production

**Figure 8:**
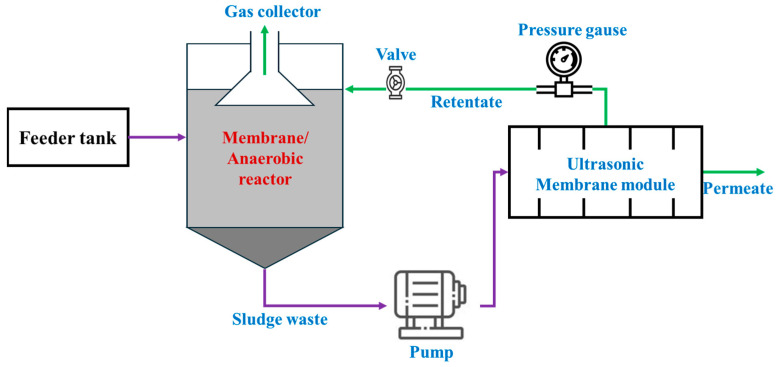
Schematic diagram illustrates integration of anaerobic process and membrane technology for improving POME treatment and enhancing biogas production. Adapted with permission from [6].

Although one of our main objectives was to review the application of membranes for the purification of biogas from POME, we attempted to summarize the integration of membrane technology with other systems to enhance biogas production in relation to the OM degradation rate. Increasing raw biogas production from POME ultimately leads to a higher yield of purified gas, which can be applied in the process, as shown in Figure 4. Hence, in this scope, several studies have focused on integrating membrane technology with other systems, such as the ultrasonicated membrane anaerobic (UMAS) and membrane anaerobic systems (MAS) [57,69]. These hybrid systems are typically designed as shown in Figure 8. Ultrasonication was integrated to enhance the breakdown of particulate matter, leading to a reduced particle size and an increased fraction of soluble matter [70]. This process helps minimize membrane fouling during cross-flow filtration, while improving membrane permeability [71]. In a study focused on the UMAS, Abdurahman et al. (2023) successfully removed 96.6–98.4% of the COD from POME with a 7.5-day hydraulic retention time (HRT). Furthermore, the CH_4_ yield fluctuated from 0.24 to 0.59 gCOD/day, with the CH_4_ content in the biogas ranging from 68.8 to 73% [69], which is consistent with previous studies showing values between 63.2–74.8% and 64.6–81% [72,73]. Additionally, biogas production increased to 0.87 L/gCOD/d when the organic loading rate (OLR) was increased to 11 kg COD/m^3^/d, although the CH_4_ percentage decreased to approximately 68% [69]. This reduction in CH_4_ percentage may be due to the promotion of acid-forming bacterial growth as the OLR increases [6,74,75]. However, membranes are more susceptible to fouling under anaerobic conditions at high mixed liquor–suspended solid (SS) concentrations because the aging of activated sludge increases its viscosity, thereby allowing foulants, such as particulates, extracellular polymeric substances, and soluble microbial products, to polarize on the membrane and form a cake layer [76,77]. In another study, an integration of a continuous stirred tank reactor (CSTR) with a tubular polyvinylidene fluoride (PVDF) ultrafiltration membrane with a 0.03 µm pore size was tested to assess the efficiency of the system in removing OM and producing biogas. After approximately 60 days, the system achieved an over 97% COD removal, and biogas production reached 0.38 Nm^3^/kg-fed COD at an HRT of 10 days and a sludge retention time of 45 days [78]. These results demonstrate that the CSTR membrane system is highly effective for POME treatment and is suitable for industrial-scale applications.

In another study, 100 µm of powdered activated carbon (PAC) was added to hybrid anaerobic membrane bioreactors (MBRs) to enhance the COD removal efficiency and biogas production. The results showed that the addition of PAC increased biogas production to 142 ± 12 mL/h, with a CH_4_ content of 57.59 ± 0.15% [79]. Small PAC particles provide a larger surface area, facilitating better biomass activity and growth rates in bioreactors. Consequently, more methanogenic bacteria can work together to convert OM into biogas at a faster rate during biodegradation [80]. In a more recent study, a UASB hollow-centered packed bed (UASB-HCPB) reactor with a 5.49 L capacity, coupled with an ultrafiltration (UF) membrane, was designed to improve POME treatment efficiency and biogas production. The UF membrane was made from PVDF with a pore size of 0.01 µm and was operated in a cross-flow mode with an inlet pressure of 0.1–0.4 MPa. Although this study is still in the design phase and has not yet been tested with real POME, the authors believe that the UASB-HCPB bioreactor, combined with the membrane, could effectively separate solid biomass from the digester suspension and recycle it back to the digester, thereby increasing the biogas yield [81].

## 4. Membrane Technology for Water Reclamation from POME

**Figure 9:**
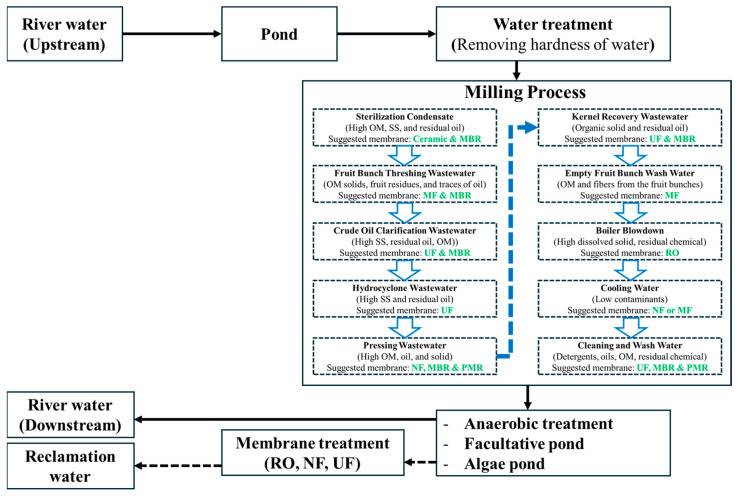
Proposed water reclamation from POME is based on traditional treatment methods, with the new concept indicated by the dotted line. In the milling process, each phase suggests the appropriate membrane to reduce contaminants, thereby decreasing the contaminant load after milling. Adapted with permission from [82,83].

Membrane technology is effective for resource recovery and wastewater treatment, particularly water reclamation, and its growth has been rapid since the early 21st century [40]. However, there are limited studies on POME water reclamation using membranes. Building on conventional POME treatment methods, we propose an innovative approach that integrates membrane technology for water reclamation [82,83], where Reverse Osmosis (RO), nanofiltration (NF), and UF are applied as the final treatment steps [84,85], as shown in Figure 9. Typically, in this figure, the membrane is integrated into each step of the milling process, following previous study proposals [83], to maximize the POME treatment efficiency.

Membrane systems commonly used in POME include MBRs, photocatalytic membrane reactors (PMRs), pressure-driven membranes, and their combinations [83]. An MBR is a hybrid technology that combines the biological treatment of activated sludge with membrane filtration [86]. Compared with conventional biological treatment, MBRs exhibited a higher efficiency in removing BOD, COD, and SSs because the removal process is based on filtration rather than gravity [87]. MBRs can be classified into three main categories: gas diffusion, filtration, and extraction [88]. These membranes have emerged as effective solutions for treating POME while generating biogas, as summarized in the previous section.

A PMR is a system that combines heterogeneous photocatalysis and membrane separation to produce chemical transformations [89]. Titanium dioxide is normally applied in PMR systems because of its excellent properties, such as a high chemical stability, high surface-to-volume ratio, low toxicity, and quantum confinement effects [90]. The main process of PMRs’ operation is the simultaneous photocatalysis of treated water while retaining the photocatalyst in the system. This feature contributes to higher efficiency, stability, and system controllability [91].

Pressure-driven membranes refer to the process of separating the permeate from the retentate with pressure as the driving force, including UF, microfiltration (MF), NF, and RO, which operate by applying pressure to separate pollutants via size exclusion [92]. According to a global market report, RO is the most widely used membrane, accounting for approximately 45% of the total [93]. UF membranes have a pore size range of 0.001–0.1 μm, and they are effective for removing colloids and smaller particles such as proteins, while allowing water and smaller solutes to pass through [94]. The pore size of the MF membranes normally fluctuates in the range of 0.1–10 μm. With this size, the MF is effective in the removal of SSs and microorganisms, while simultaneously facilitating the permeation of smaller molecules and ions [95]. The NF membranes had a pore size range of 0.001–0.01 μm [96]. This indicates that the NF membranes were near the range required for the removal of small ions. The mechanism of NF removal is not purely filtration, as with UF membranes, but also osmotic. This makes them true hybrids, bridging UF and RO membranes within the range of membrane treatment options [97]. RO membranes, with pore diameters smaller than 0.001 μm, achieve high rejection rates for organic compounds, dissolved salts, and various pollutants, delivering purified water suitable for discharge or reuse [98]. In addition to their high removal rates, RO membranes are highly resistant to biological degradation and high temperatures and are mechanically stable [99].

Most studies on membrane applications for water reclamation from POME have primarily focused on pressure-driven membrane processes. These processes use pressure as a driving force to create a permeate flux across the membrane that moves from the bulk solution to the permeate side [100]. This category includes the MF, UF, NF, and RO membranes. Notably, combining biological treatments with UF, NF, and RO membranes has yielded substantial results for POME water reclamation [101,102].

A key study on the use of reclaimed water from POME as drinking water was conducted by Ahmad et al. [85], who used UF and RO membranes as the final step after pretreating POME with electrocoagulation and flocculation. This process successfully recovered 78% of drinking water from POME. In the study, the transmembrane pressure of the UF was maintained at 2 bar, whereas that of the RO was maintained at 45 bar. Although the water quality (e.g., COD, TDS, turbidity, and color) met the USEPA drinking water standards, both membranes experienced a decline in permeate flux rates. UF membranes are particularly susceptible to fouling because of their increased resistance to cake layer formation [12]. In addition, internal fouling poses a significant challenge that often surpasses the impact of surface cake formation. This issue is largely influenced by pore surface density and size distribution [103,104]. This occurs when OM, proteins, colloids, fine particles, and microbial byproducts infiltrate and accumulate within membrane pores, leading to a reduced filtration efficiency [103]. Although UF shows potential for POME water reclamation, addressing fouling requires a detailed strategy. To minimize membrane fouling and ensure continuous operation, a substantial amount of water is required for membrane cleaning [105]. Typically, internal fouling is more difficult to remove than external fouling through simple flow reversal. Therefore, a combined approach using back-pulsing and surface modification has been suggested as an effective strategy for mitigating cake layer formation and internal pore blockage [103,106].

To date, most studies on water reclamation from POME have focused on the production of boiler feedwater, as shown in Table 3. In these studies, UF membranes were the most commonly used for water reclamation. In early studies on this topic, the authors integrated UF and RO membranes following coagulation, sedimentation, and adsorption as pretreatment processes [107]. One of these studies used a UF membrane with a 0.36 m^2^ area, 0.5–1.0 µm pore size, and 0–7 bar operating pressure, followed by a TFC-type tubular RO membrane with a 0.9 m^2^ area and 0–60 bar pressure. The results showed an approximately 99% removal of COD and BOD and almost 100% removal of turbidity from POME. In another study, UF was used as a potential membrane for POME water reclamation for boiler feed water but with different operating conditions and an emphasis on addressing fouling issues. This was achieved by improving the pretreatment efficiency and using chemicals for membrane cleaning [102,108]. Similarly, Amosa et al. used 60 g/L PAC for adsorption in upstream biotreated POME (BPOME) for 60 min. After each filtration, a 1N NaOH solution at 50 °C was used to clean the membrane. The results indicated that the use of PAC for pretreatment and cleaning effectively restored the integrity of the membrane to approximately 100% of its initial active strength. Similar results and conclusions have been reported in previous studies [109,110].

In addition to RO and UF membranes, forward osmosis (FO) has attracted attention as a promising alternative because of its low energy consumption, minimal operational pressure, simple equipment requirements, and reduced fouling [111,112]. FO has been applied successfully in treating various types of wastewater, including municipal wastewater, oily and drilling wastewater, landfill leachate, and even simulated radioactive wastewater, and so on [111]. However, FO is less attractive than UF or RO for treating POME. This is primarily because FO requires energy-intensive draw solution (DS) regeneration for water recovery and solute reuse, which adds to the operational complexity and costs, particularly in high-volume POME treatment [113]. Although FO generally exhibits better fouling reversibility than pressure-driven membranes, the complex composition of POME can still cause membrane clogging and reduce permeability over time. Additionally, the high calcium content in POME [114] can permeate the DS, leading to severe fouling and a significantly decreased permeate flux [115,116]. Because of such challenges, research on FO for POME treatment and water reclamation remains limited. However, a foundational study by Arifin et al. (2015) explored the feasibility of FO for POME treatment and demonstrated that higher temperatures enhance water flux. Their findings showed that water flux increased by 7–9% when the temperature rose from 25 °C to 35 °C, and by 32–75% when increased from 25 °C to 45 °C [117]. The results suggest that an improved water flux promotes dynamic flow, mitigates concentration polarization, and reduces organic deposition, biofouling, and scaling. Furthermore, a higher flux enhances the system efficiency by accelerating water recovery and reducing the membrane cleaning frequency, offering partial support for understanding FO fouling behavior in POME treatment.

**Table 3:** Summary of research on the application of membranes for water reclamation from POME.

POME	Pretreatment	Type of Membrane/Operation Parameters/Operation Mode	Performance Efficiency	Cleaning Frequency (Hours)	Reuse Application	Refs.
POME: COD = 50,000 mg/L, TDS = 20,500 mg/L, turbidity = 11,000 NTU, and oil and grease = 4000 mg/L	Coagulation and flocculation	UF/pressure 2 bar and RO/pressure 45 bar/pilot plant	COD = 88 mg/L, TDS = 130 mg/L, turbidity, NTU = 0.02. Color, odor, turbidity, and oil and grease were completely removed at a pH of 6.63	24	Drinking water set by USEPA	[85]
Aerobic digester POME with dilution: COD = 152 mg/L, TDS = 394.68 mg/L, and turbidity = 18.1 NTU	No applications	NF (NF270) with a water permeability coefficient of 21.18 L/m^2^/h, and RO (XLE and BW30) with water permeability coefficients of 14.15 L/m^2^/h and 6.54 L/m^2^/h, respectively/bench scale	NF270: COD = 8 mg/L, TDS = 24 mg/L. XLE: COD = 5 mg/L, TDS = 21.1 mg/L BW30: COD = 5 mg/L, TDS = 21.5 mg/L	6	Boiler feed water set by USEPA	[84]
POME: COD = 43,155 mg/L, SS = 18,975 mg/L, oil and grease = 3172 mg/L	Natural coagulant, latex adsorption and activated carbon	UF (ceramic and PVDF)/pilot plant	Ceramic membrane: COD, SS, and oil and grease = 5.767, 0.27, and 1.22 mg/L, respectively. PVDF: COD = 258 mg/L, SS and oil and grease are completely removed.	No mention	Drinking water set by USEPA	[118]
BPOME: COD = 1387 mg/L, TDS = 970 mg/L, turbidity NTU = 840	PAC adsorption	MF/0.1 µm, 40 kPa/bench scale	COD = 92 mg/L, TDS = 760 mg/L, turbidity NTU = 5	1	Irrigation water	[119]
BPOME: COD = 1387 mg/L, TDS = 970 mg/L, and turbidity NTU = 840	PAC adsorption	UF (polyethersulfone material), effective filtration area: 0.1 m^2^/bench scale	COD = 4 mg/L, TDS = 380 mg/L, and turbidity NTU < 1	1	Boiler feed water set by USEPA	[120]
POME: BOD = 2700 mg/L turbidity NTU = 8124 SS = 5709 mg/L	Coagulation and flocculation	UF/5 bar and RO/30 bar/pilot plant	UF: BOD = 390 mg/L, turbidity NTU = 1.08, SS = 505.74 mg/L RO: BOD = 30 mg/L, turbidity NTU = 0.05, SS = 198.17 mg/L	1	WHO water reuse standard	[121]
POME: COD = 26,107 mg/L, BOD = 15,800 mg/L, turbidity NTU = 10,563	Coagulation, sedimentation, and adsorption	UF (0.5–1 µm, 0–7 bar) and TFC-type tubular RO (0–60 bar)/pilot plant	COD = 314 mg/L; BOD = 91 mg/L; yurbidity NTU = 0.81	No mention	Boiler feed water	[107]
POME: Oil and grease = 1077–7582 mg/L	Biological treatment	UF (2 bar) and RO (13 bar)/pilot scale	Completely remove oil and grease	No mention	Boiler feed water	[108]
POME: COD = 75,000 mg/L, BOD = 27,000 mg/L, SS = 50,000 mg/L	Biological treatment	UF (2 bar) and RO (13 bar)/pilot scale	The effluents COD, BOD, and SS were not detected.	No mention	Boiler feed water	[102]
POME at the point before discharging to river COD = 170.8 mg/L, BOD = 97 mg/L, SS = 43 mg/L, turbidity NTU = 17.6	No application	UF (PEC and RC)/bench scale	COD = 48 mg/L, BOD = 21.8 mg/L, SS < 25 mg/L, turbidity NTU = 0.72	No mention	Recycling of mill follows WHO water reuse standards.	[122]

BPOME, biotreated POME; PAC, powdered activated carbon; XLE, extra low-energy RO membrane; BW30, brackish water membrane; PVDF, polyvinylidene fluoride; TFC, thin-film composite; RC, regenerated cellulose; PES, polyethersulfone.

## 5. Membrane Technology in MFCs for Electricity Recovery from POME

MFCs are bioelectrochemical systems that have developed rapidly over the past few decades, and they are regarded as a potential technology for extracting renewable resources from wastewater. A typical MFC system consists of anode and cathode compartments separated by a membrane [123]. Furthermore, MFCs harness electricity from microorganisms during wastewater treatment [124]. The major advantage of MFCs is their ability to use various organic materials such as wastewater, agricultural waste, and industrial byproducts, which renders them a potential solution for sustainable energy production and wastewater treatment. Recent research has shown that MFCs can achieve impressive power densities, exceeding 4200 mW/m^3^ after the complete removal of contaminants and COD [125]. Owing to such advantages, MFCs are considered a potential technology for POME applications. Recent studies have demonstrated the feasibility of using MFCs to generate bioelectricity while simultaneously treating POME (Table 4).

Although MFCs have several advantages, they have certain limitations that should be addressed before they can be fully implemented as a technology in future. One crucial issue is that MFCs do not achieve an ideal performance owing to factors such as a short lifespan, low production rates [126], limited efficiency [127,128], and membrane fouling [129]. Such limitations are influenced by anode, cathode, and membrane components [130].

**Figure 10:**
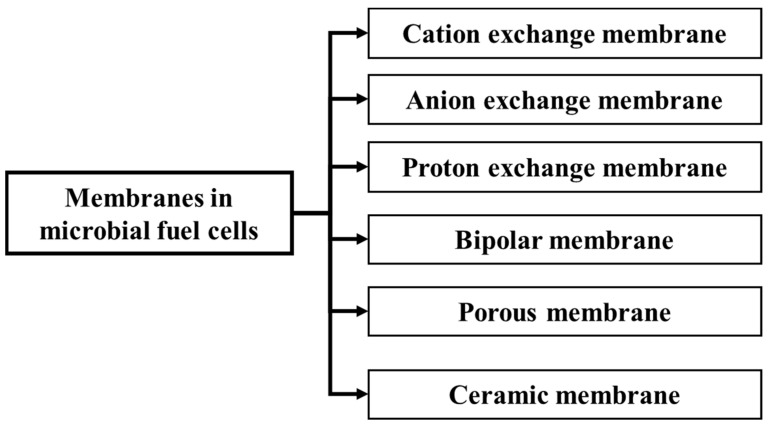
Most commonly used membrane in MFC system. The figure was constructed based on data reported in [139].

For anodes, significant progress has been achieved with the introduction of brush electrodes and graphite fibers [131]. Recent studies have identified potential alternatives to precious metals by exploring transition metals and other cost-effective metal compounds as viable cathode catalysts [132]. These advancements are crucial for improving the efficiency and reducing the cost of MFCs. However, the widespread application of MFCs is challenging because of the high cost of the membranes and their impact on performance [131]. Therefore, membrane selection should consider factors such as internal resistance, substrate loss, biofouling, and oxygen diffusion [133]. Figure 10 summarizes the most commonly used membrane types for MFCs. To address such challenges, researchers have explored alternative materials, such as sulfonated polymers, which are commonly used in MFC systems for POME treatment, to enhance the performance of Nafion membranes [134]. For example, Zhao et al. demonstrated that composite membranes composed of sulfonated poly (ether ketone) and nanofillers outperformed Nafion membranes [135]. Ceramic and fiber membranes have also been considered potential ion exchangers in MFCs [131]. Despite progress in membrane development, challenges have been reported, particularly proton transfer limitations and the risk of oxygen leakage, which contribute to increased internal resistance and reduced MFC performance, thereby limiting their practical applications [136,137]. Consequently, researchers should continue to identify optimal membrane separators for MFCs. Ideal membranes should have a low cost, minimal internal resistance, selectivity, chemical and mechanical stability, and fouling resistance characteristics [138].

In most studies on the application of MFCs for POME, proton exchange membranes (PEMs) such as Nafion have been commonly used as the primary separator, as shown in Table 4. This is likely because the PEM allows efficient proton transfer between the anode and cathode compartments, which is crucial for maintaining a consistent electron flow and enhancing power generation [130]. In contrast to the 304 mW/m^3^ [140] and 1236 mW/m^3^ [141] reported in other studies, a study by M. Amirul Islam et al. [142] achieved the highest power output of 1667 mW/m^3^ among the MFCs that use PEMs. In their study, the authors used a carbon brush and PACF as the anode and cathode, respectively, and Nafion as the PEM. To enhance power generation, wild-type *Klebsiella variicola* was introduced as an efficient inoculum for anodes in POME-operated MFCs. Similarly, Jong et al. [143] used diluted POME with a COD of 200 mg/L to achieve a maximum power density of 62.2 W/m^3^.

To the best of our knowledge, only one study has applied an anion-exchange membrane (AEM) to MFC systems. This study provides a new route for MFCs’ application in POME, potentially shifting the conventional use of PEMs. The results of this study demonstrated a relatively high power density generation of 180 mW/m^2^ [144] with an influent COD of 1000 mg/L, compared to 22 mW/m^2^ [145], 45 mW/m^2^ [140], and 38.38 mW/m^2^ using a cation exchange membrane (CEM) [146].

Generally, POME with high COD concentrations negatively affects MFC performance, leading to reduced electricity generation. This may be because of excessive OM, which can inhibit the metabolism of electrochemically active bacteria, thereby reducing their electron generation efficiency. Additionally, a high organic content may cause excessive biofilm formation on the anode surface, thereby increasing the internal resistance and obstructing electron transfer. This overgrowth presents operational challenges and reduces electricity generation. Therefore, to optimize MFC performance, it is crucial to reduce the influent COD value, potentially through integration with pretreatment technologies aimed at lowering the OM content of raw POME.

**Table 4:** Summary of research on application of MFCs for electricity recovery from POME.

Type of POME	Type of MFCs	Anode Material	Cathode Material	Membrane	Power Density/Volumetric Power Density/Voltage	Refs
POME + anaerobic sludge (COD = 34,180 mg/L)	Single-chamber air cathode	Carbon brush	PACF	PEM	1667 mWm^−3^	[142]
POME + anaerobic sludge (COD = 60,600 mg/L)	Two-compartment MFC	PACF	PACF	PEM	45 mWm^−2^/304 mWm^−3^	[140]
POME (COD = 589 mg/L)	Two-compartment MFC	Carbon paper	Carbon paper	PEM	0.444 V	[147]
POME (COD = 68,360 mg/L)	Air cathode, single chamber	Carbon brush	PACF	PEM	1236 mWm^−3^	[141]
POME (COD = 98,000 mg/L)	Two-compartment MFC	Carbon rod	Carbon rod	CEM	3.21 mWm^−2^	[148]
POME from anaerobic tank (COD = 1000 mg/L)	Tubular configuration with an air cathode	Carbon brush + PDDMAC and PTFE	Carbon cloth with activate carbon	AEM	180 mWm^−2^	[144]
POME (COD = 66,133 mg/L)	Air cathode double-chambered MFC	Pretreated carbon cloth	Pretreated carbon cloth	CEM	38.38 mWm^−2^	[146]
Diluted POME (COD = 1000 mg/L)	Two cylindrical compartments	PACF	PACF	PEM	22 mWm^−2^	[145]
POME (COD = 49,590 mg/L)	Two-compartment MFC	Carbon electrode	Carbon electrode	CEM	625 mV	[149]

PACF, polyacrylonitrile carbon felt; PDDMAC, diallyldimethylammonium chloride; PTFE, polytetrafluoroethylene; PEM, proton exchange membrane; Refs, references.

## 6. Sustainability of POME Valuable Resources, Challenging, and Membrane Perspective

Among the potential POME resources, biogas is the most valuable. Biogas produced from POME is a potential renewable energy source that addresses environmental and energy challenges, while remaining consistent with the principles of a circular economy. POME, which has abundant biodegradable OM, supports anaerobic digestion, resulting in biogas primarily composed of CH_4_ and CO_2_. The continuously operating oil palm mills in palm oil-producing countries [57], such as Indonesia, Malaysia, Thailand, Colombia, and Nigeria, are potential suppliers of raw materials for biogas production. This ongoing production of biogas could provide significant benefits to these countries if harnessed for energy, while contributing to the reduction of GHG emissions. For instance, in Malaysia, if all POME were treated anaerobically, approximately 500,000 t of CH_4_ could be produced [150]. This amount of CH_4_ is equivalent to approximately 800 million L of diesel in terms of CV and can generate an estimated 3.2 million MWh of electricity, which is equivalent to a 400 MW power plant operating at 40% efficiency [150]. Power generated from POME-derived biogas could potentially supply electricity to 700,000 households in Malaysia [150]. However, raw biogas from POME often contains impurities, such as CO_2_, H_2_S, and moisture, which reduce its CV and complicate its direct use. Therefore, the development of cost-effective, high-performance technologies for purifying and upgrading biogas from POME is essential to address environmental and economic challenges.

The use of MFCs to address the POME issue while generating electricity introduces a novel approach to the global shift toward a circular economy. With this dual function, MFCs are an energy-efficient alternative to conventional POME treatment methods such as ponding systems or anaerobic reactors, which often require additional energy inputs and generate GHG emissions [3]. Additionally, MFCs can reduce sludge production, minimize chemical usage, and provide odor control owing to their anaerobic operation [151]. However, high concentrations of pollutants, primarily OM, can hinder the effectiveness of MFCs for POME treatment. Therefore, integrating MFCs with pretreatment technologies, such as biogas reactors, is essential for breaking down OM before it enters the MFCs, thereby enabling CH_4_ recovery during OM degradation. By incorporating the MFC technology into POME treatment processes, industries can achieve sustainable wastewater management while recovering bioenergy, which is consistent with the goals of renewable energy generation and environmental sustainability [136].

The use of POME membranes for recovering valuable resources, particularly for purifying and upgrading biogas, has significantly increased, and it has become one of the most widely adopted technologies in new installations. This is due to their high CH_4_ recovery rates and minimal CH_4_ losses (<5% *v*/*v*), with their low energy consumption (0.2–0.38 kWh/Nm^3^). Additionally, membranes do not require the addition of chemicals throughout the process, thereby avoiding the production of toxic byproducts, which is a crucial advantage [152]. Membrane technology is highly scalable because multiple membrane modules can be integrated into existing installations and adapted to various process configurations [153]. Furthermore, membranes can achieve a high biogas purification, typically ranging from 90 to 99%, depending on the membrane type and structure. For example, polyether ether ketone membranes can achieve a CH_4_ concentration of 98% *v*/*v* at a feeding pressure of 25 bar [154,155]. Cellulose acetate membranes require feeding pressures of ≥40 bar and can achieve CH_4_ recovery yields of ≥90% [156]. Additionally, PSf membranes can achieve a 95–98.8% *v*/*v* CH_4_ recovery at feeding pressures of 6–10 bar [157], whereas polyester carbonate (PEC) membranes can achieve a 96% *v*/*v* CH_4_ recovery at a feeding pressure of 7 bar [158]. Interestingly, some studies have demonstrated that membrane modules made from PSf, Polyimide, and PEC can remove H_2_S from biogas with efficiencies ranging from 55% to 89% [158,159,160]. Despite these advantages, membranes have higher initial investment costs than other technologies such as pressure swing adsorption, water, chemicals, and organic scrubbing. For smaller facilities (<500 Nm^3^/h), the membrane investment costs are relatively high, ranging from EUR 4700 to 6000/(Nm^3^/h). However, for larger capacities, the investment cost becomes comparable to that of other technologies, with reported costs ranging from 1300 to 2700 EUR/(Nm^3^/h) [161,162,163]. Although the initial investment is high, maintenance costs during membrane operation are low, typically accounting for 3–4% of the initial investment, rendering it a viable long-term solution [164].

Membrane technology has emerged as a potential solution for POME reclamation, significantly reducing the need for external water intake during milling and minimizing the environmental impacts [107]. Membrane processes, such as MF, UF, NF, and RO, are highly efficient in removing various contaminants from POME, such as SS, OM, and dissolved salts [165,166]. Owing to its flexibility, this technology can be applied to both small- and large-scale palm oil mills, while consistently meeting stringent effluent discharge regulations for water resources. Despite its numerous advantages in separation processes, such as gas and water reclamation, several critical challenges hinder its widespread application, such as membrane fouling, limited selectivity, and the trade-off between permeability and selectivity. Therefore, transitioning membrane technology from a potential solution to a fully practical solution for POME treatment is challenging [167]. To address these barriers, advancements in membrane materials, optimized pore sizes and structures, hybrid membrane approaches, and improved design strategies are required to enhance their durability and long-term stability. Once these limitations are addressed, membrane technology will become the ideal choice for POME treatment, contributing to improved separation processes and enhanced electricity generation in MFCs.

Scaling up membrane technology for POME treatment presents significant challenges, primarily due to membrane fouling. High concentrations of OM, oil, and grease reduce the filtration efficiency, necessitating frequent cleaning or membrane replacement [168]. Among the various fouling mechanisms, cake filtration is the most prevalent, because particulate materials such as fibers, cell debris, and other impurities accumulate on the membrane surface and within its pores, exacerbating fouling issues [169]. The formation of impermeable layers on membrane surfaces can reduce the flux by up to 19.45% at higher pressures (50–200 kPa) [170]. An increased applied pressure can also lead to the consolidation of foulant layers, potentially causing irreversible membrane fouling over time [171]. Additionally, POME contains minerals and salts such as calcium carbonate, calcium phosphate, and magnesium sulfate, which form solid deposits on the membrane surface. This scaling effect significantly reduces membrane permeability and hinders fluid flow, thus diminishing the overall efficiency of the separation process [172]. Another critical challenge is biological fouling, as microorganisms such as bacteria and fungi colonize the membrane surface and form biofilms. These biofilms not only obstruct filtration but also act as adhesive matrices, trapping additional OM and particulates, further intensifying the fouling and reducing the membrane’s performance [169]. This challenge also affects the performance of MFCs, where microorganisms typically depend on membrane surfaces for growth [173]. Severe issues have been observed, resulting in a substantial decline in output current [174]. Over time, biofouling-related issues necessitate frequent membrane replacement, increasing operational costs and limiting the economic feasibility of large-scale applications [175]. Addressing these challenges requires the development of advanced membrane materials, effective pretreatment strategies, and cost-effective operational approaches. Innovation in these areas is essential for the long-term viability and sustainability of membrane-based POME treatment, enabling a broader adoption of the technology in industrial-scale applications.

## 7. Conclusions

The palm oil industry is expected to continue expanding, particularly in major oil-producing countries, such as Indonesia and Malaysia. Consequently, POME presents a major opportunity as a sustainable source for biogas production, electricity generation, and water recycling. As an abundant and cost-free feedstock available in palm oil mills, POME ensures a steady supply of substrates for valuable applications without additional expenses. In the case of biogas as a renewable energy source, purification and upgrading are essential. Currently, biogas upgrading applications in Asia, particularly Southeast Asia, are limited and often partially funded by governments, rendering them commercially unfeasible. Currently, CH_4_ production from POME is still below expectations, as many palm oil mills are reluctant to adopt higher-efficiency technologies, such as closed anaerobic digester tanks, owing to the lower operating costs and ease of operation of open anaerobic digesters. This wastes resources and contributes to global warming through CH_4_ and CO_2_ emissions. Therefore, biogas recovery from POME has significant economic and environmental benefits. To maximize these benefits, anaerobic technology must be improved to convert the OM in POME into valuable CH_4_ gas. Once produced, purification and upgrading technologies should be used to enhance the value of POME-derived biogas before it can be used as a renewable energy source.

Additionally, large volumes of water are used during the extraction of CPO from fresh fruit bunches, and approximately 50% of this water is converted to POME. Currently, most of this wastewater is treated using traditional open pond systems, with a small percentage being reclaimed for palm tree irrigation rather than for other uses, such as drinking water. MFCs have recently emerged as a novel approach for generating electricity from POME; however, research on this method is insufficient. However, this subject has increasingly attracted attention, owing to the recognition of the valuable resources contained in POME. Advanced technologies are being explored to promote a circular economy and reduce the environmental impacts. Membrane technology is considered an ideal solution because of its high separation efficiency, ability to prevent toxic byproducts, and cost-effective long-term operation. However, this technology has certain limitations that must be addressed to transition from a potential concept to a practical solution for POME treatment.

## Figures and Tables

**Table 1:** Typical concentration of raw POME wastewater and standard discharge limits set by Malaysian department of environment (DOE).

Parameters ^(a)^	Concentration	Standard Discharge Limits ^(b)^	References
pH	3.4–5.2	5.0–9.0	[11]
BOD	10,250–43,750	20	[11]
COD	15,000–100,000	NA	[11]
Turbidity, NTU	17,000	NA	[12]
Color, ADMI	>500	100	[13]
Total solids	40,500–75,000	200	[14]
Total volatile solids	9000–72,000	NA	[15]
Grease and oil	130–18,000	5	[16]
Ammonium–nitrogen	25–35	NA	[17]
Total nitrogen (TN)	180–1400	150	[18]
Total phosphorous (TP)	95–120	NA	[19]
Alkalinity	100–150	NA	[17]
Total carbohydrate	16,200–20,000	NA	[17]
Pectin	3400	NA	[3]
Lignin	4700	NA	[3]
Phenol	5800	NA	[20]
Carotene	8	NA	[3]
Potassium (K)	1281–1928	NA	[13]
Magnesium (Mg)	254–344	NA	[13]
Calcium (Ca)	276–405	NA	[13]
Boron (B)	7–8	NA	[21]

^(a)^ All values are in mg/L, except pH, turbidity, and color. ^(b)^ Standard discharge limits in place since 2015. NA is not available.

**Table 2:** Percentage concentration of each component of biogas after the purification process is shown in this table. Notably, MTR refers to the membrane modules, which contain composite spiral-wound membranes with a selective layer, whereas S and D denote single-stage and double-stage membrane processes, respectively. The table was constructed based on data reported in [64].

Parameters	Units	Input		Retentate		Permeate	
		S-MTR	D-MTR	S-MTR	D-MTR	S-MTR	D-MTR
Mass Flow	kg/h	1828.31	1828.31	1184.38	1184.38	643.41	643.41
Pressure	bar	1.0342	13.7	1.1031	0.6	14.8754	0.9
CO_2_	%	40	9.1	90.7871	75.3	9.2129	**0.2**
CH_4_	%	58.999	90.2	7.4464	3.1	**90.2486**	**98.9**
H_2_S	ppm	-	1	-	1	-	**1**

## Data Availability

No new data were created or analyzed in this study. Data sharing is not applicable to this study.
